# Saliva as a medium to detect and measure biomarkers related to pain

**DOI:** 10.1038/s41598-018-21131-4

**Published:** 2018-02-19

**Authors:** Hajer Jasim, Anders Carlsson, Britt Hedenberg-Magnusson, Bijar Ghafouri, Malin Ernberg

**Affiliations:** 1 Division of Oral Diagnostics and Rehabilitation, Department of Dental Medicine and Scandinavian Center for Orofacial neuroscience (SCON), Karolinska Institutet, SE14104 Huddinge, Sweden; 2 Folktandvården Stockholm AB, Stockholm, Sweden; 3 Division of Community Medicine, Department of Medical and Health Sciences, Faculty of Health Sciences, Linköping University and Pain and Rehabilitation Center, Anaesthetics, Operations and Specialty Surgery Center, Region Östergötland, Linköping, Sweden

## Abstract

Saliva is often neglected as a body fluid of diagnostic or prognostic value, even though generally well accepted by the patients. This is due to lack of a standardized collection procedure. The aim of this study was to identify the ideal saliva collection technique and develop new sensitive methods to detect and analyse markers related to pain in healthy pain-free subjects. Plasma and five different saliva collection approached was evaluated during strictly controlled conditions. Levels of nerve growth factor (NGF), calcitonin gene-related peptide (CGRP) and brain derived neurotropic factor (BDNF) were determined using novel western blotting based technology. Glutamate and substance P (SP) was determined using commercial available methods. Several new isoforms were found for NGF, CGRP and BDNF in saliva. The isoform pattern showed significant variation in both expression and chemiluminescence levels between different collection methods. New sensitive methods to study pain related markers in saliva were developed in this study. Furthermore, we are first to demonstrate a correlation between the Glutamate concentration in stimulated whole saliva and blood. However, the fundamental conclusion drawn is the importance of consistency in the collection method.

## Introduction

During the past decades, salivary diagnostics have received increasing attention. The salivary glands are integrated into the neuroendocrine system and contains a wide array of biomarkers that might play important roles in the pathophysiology of chronic pain conditions^1^.

It was recently reported that the intramuscular concentration of glutamate, which has a significant role in nociceptive processing, was elevated in chronic myalgia and associated to pain sensitivity^2–4^. Nerve growth factor (NGF) is a neuropeptide that facilitates neuronal regeneration and acts as a protective factor for neurons. NGF seems to play an important role in hyperalgesia and its concentration increases during inflammation and is up-regulated in response to noxious stimuli^5^. Calcitonin gene-related peptide (CGRP), brain derived neurotropic factor (BDNF), and substance P (SP) are other examples of abundant neuropeptides in nervous tissue. These play important roles in the development of pain and hyperalgesia. A role for CGRP and BDNF has been implicated in migraine and headache based on its increased saliva and plasma concentration during active pain periods^6–9^.There is also evidence that salivary SP levels increases with noxious stimulation, indicating that SP may play a role in central sensitization associated with chronic pain^7,10^.

These findings indicate that on-going activity in sensory neurons may be reflected in the change of the peripheral neuropeptide concentration. However, most of the past studies on neuropeptides in chronic pain have assessed plasma, cerebral or interstitial concentrations. Only a few studies have investigated the levels in saliva^7–9,11^. Many substances enters saliva from the blood by passing through the intercellular spaces by transcellular or paracellular diffusion^12^. As a result, most substances found in blood are also present in saliva. Therefore saliva is functionally equivalent to serum in reflecting the physiological state of the body. Saliva collection provides several advantages over blood^13^, such as being easy and non-invasive. There are therefore compelling reasons for exploring saliva as a diagnostic and prognostic fluid in pain research^14,15^. The few studies that have measured neuropeptides in saliva present limitations regarding the collection and analysis of the samples^1,16^. Whole saliva is not a single fluid, but rather a complex mixture of the secretions from the salivary glands. The relative contribution of the different glands to whole saliva depends on the method of collection, the degree of stimulation, age and even the time of day^17^. This suggests that different approaches may have to be adopted when studying putative pain biomarkers in saliva.

It is obvious that there is potential value in measuring salivary biomarkers as a diagnostic tool and an objective approach to study pain. However, there is a need of evaluating different collection methods and develop more sensitive techniques for analysis. The aims of this study were to evaluate the concentration of NGF, CGRP, BDNF, glutamate and SP and their relation to plasma in saliva collected with different methods, and to develop new sensitive technology to study salivary NGF, CGRP and BDNF and different isoforms of these.

## Materials and Methods

### Participants

Twenty healthy participants, ten men and ten age-matched women, with a mean ± SD age of 24.8 ± 3.1 years where included in the study.

Inclusion criteria were good general health, age ≥18 years, and body mass index <30 kg/m^2^. Participants had also to be free of fever/or cold and maintain exceptional oral hygiene on the day of collection.

Exclusion criteria’s were any current pain, diagnosed systemic muscular or joint diseases, such as fibromyalgia, clinical signs of temporomandibular disorders (TMD), rheumatoid arthritis, whiplash-associated disorder, neurological disorders, pregnancy or lactation, high blood pressure, tobacco usage, regular use of medications including oral contraceptives, use of antidepressants or analgesics during the last week, and oral complaints, such as oral dryness or mucosal lesions. If dental examination revealed less than 22 teeth or extensive prosthodontics rehabilitations, insufficient oral hygiene, hyposalivation, oral diseases, mucosal lesions or extensive tooth wear participant were excluded from further involvement in the study. Participants reporting elevated perceived levels of psychological distress were also excluded.

All participants were requested not to eat, drink or brush their teeth 1 h prior to the trial, and not consume any alcoholic beverages 24 h prior to collection. They were also instructed to avoid dietary products high in tryptophan content, and keep a detailed food log 24 h prior to collection. The participants arrived to the clinic in the early morning. Prior to sample collection a brief interview was carried out by the examiner to ensure that they had followed the instructions, which all had. In the next step they were instructed to fill in validated questionnaires and then a clinical examination was performed as described below.

According to the power calculation, inclusion of 20 participants would be sufficient to detect a statistically significant difference of 20% (SD 30%) in biomarker level between samples with a power of 80% at a significance level of 5%.

All participants received information regarding the objectives and procedures of the study and gave their informed written consent before the start of the study. The study was approved by the Regional Ethical Review Board in Stockholm, Sweden (2014/17–31/3) and followed the guidelines according to the Declaration of Helsinki.

### Questionnaires and clinical examination

The Diagnostic Criteria for TMD (DC/TMD)^18^ was used as a screening instrument for identification of participants with TMD signs that may not be presented during the interview and general examination.

In addition to the DC/TMD examination the participants underwent a general dental examination including inspection of the oral mucosa, gums, teeth, salivary glands, and occlusion.

The Patient Health Questionnaire (PHQ-9 and PHQ-15), the Perceived Stress Scale-10 (PSS-10), the Generalized Anxiety Disorder scale (GAD-7), and Jaw Functional Limitation Scale (JFLS) included in the DC/TMD questionnaire^13^ were used as screening instrument to measure symptoms of depression, somatization, stress, anxiety and jaw function.

### Saliva collection

Prior to saliva collection participants were instructed to rinse their mouth with distilled deionized water in order to remove debris and moisturise. Saliva collection started after 10 minutes of rest. In between every sampling, participants were instructed to rest for 15 minutes to neutralize salivary flow. During the collection, participants were asked keep their eyes open during salvation and not to speak or mentally stimulate salivary flow.

All saliva samples were collected in the same order, in the same clinical room and at the same time, between 8:30 and 10:30 am. To prevent degradation of sensitive peptides all samples were collected on ice in precooled polypropylene tubes. Immediately after collection a Protease Inhibitor Cocktail (Sigma Aldrich v/v 1:500) was added. All samples were then centrifuged at 700 × g for 15 minutes at 4 °C to remove debris. The supernatant (upper 2/3) of each sample was fractionated into 100 µl aliquots and frozen at −70 °C until analyses.

**Figure 1:**
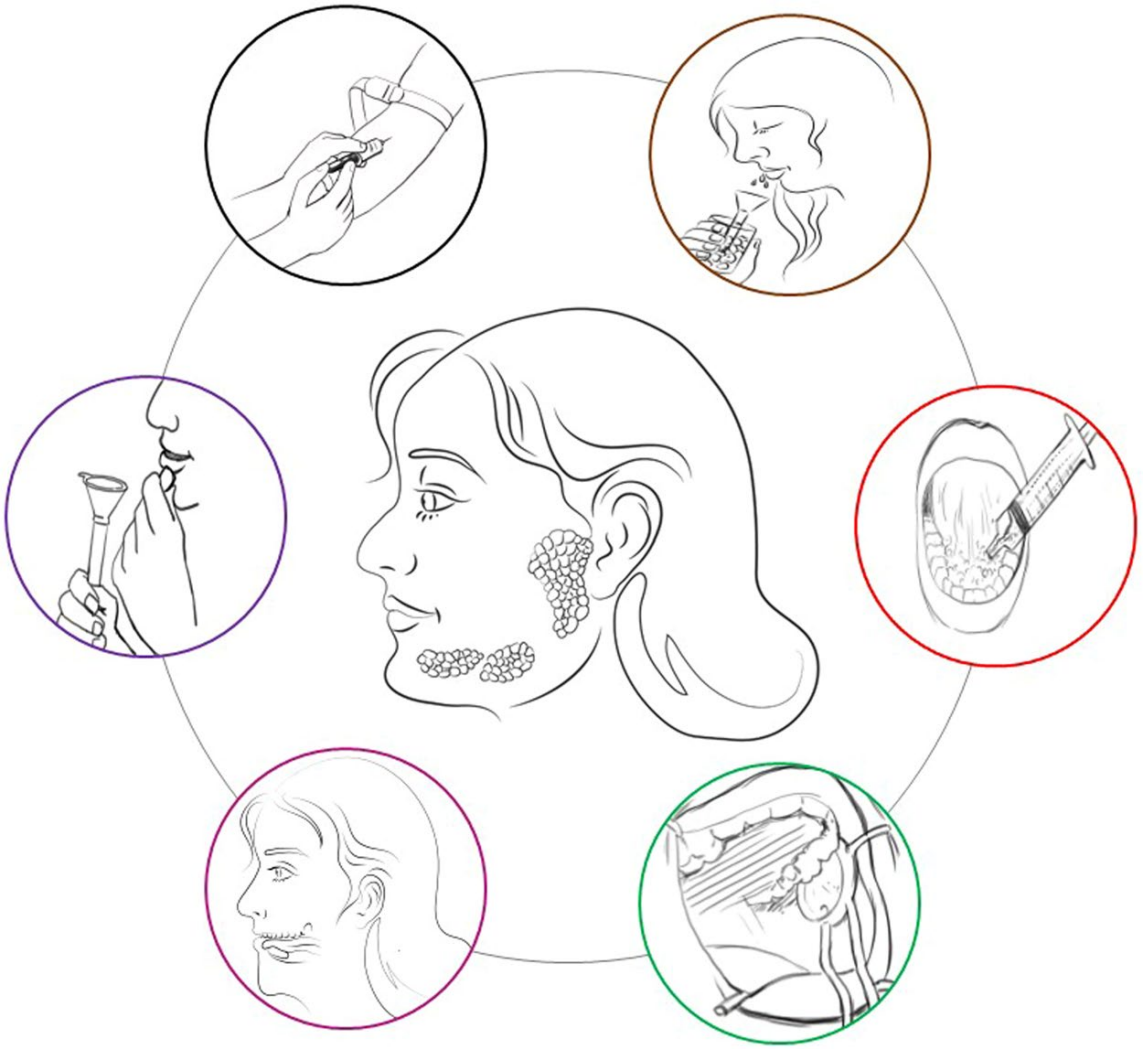
Illustrative overview of the main salivary glands and different collection approaches used in the study. The colours are associated with the different collection methods explained in the diagrams.

A total of five different saliva sampling techniques were evaluated (Fig. 1) as earlier described by Jasim *et al*., 2016^13^.

#### Unstimulated whole saliva

Participants was instructed to sit upright and with their head slightly titled forward allow saliva to collect on the floor of the mouth and dribble into a 5 ml polypropylene tube,

#### Unstimulated sublingual saliva

While blocking the Stensen’s duct, sublingual saliva was collected from the floor of the mouth with a syringe every second minute. Samples from the first 2 minutes were discarded.

#### Stimulated parotid saliva

Pure parotid saliva was collected using a modified Carlsson-Critten collector while actively stimulating salivary flow with citric acid solution as earlier described by Jasim *et al*., 2016.

#### Stimulated sublingual saliva

Saliva Bio Oral Swab^®^ (Salimetrics) was placed for around 2 minutes under the tongue while stimulating with 2% Citric acid until the swab was fully covered in saliva. The fluid was then obtained by centrifugation.

#### Stimulated whole saliva

Saliva was stimulated by chewing on paraffin tablets (Orion Diagnostica, Finland) as described earlier by Jasim *et al*., 2016.

### Blood collection

After the last saliva sample, venous blood samples were collected in 8.5 ml EDTA PPT tubes (BD, California, US) from all subjects. The sample was mixed gently for 1 minute and then immediately placed on ice for 30 minutes. The samples were then centrifuged at 1000 × g for 15 minutes at 4 °C, and the upper 2/3 of the plasma was stored as aliquots at −70 °C until analysis.

### Glutamate quantification

The concentration of glutamate was determined essentially as described previously^19^. Briefly, 20 µl saliva and plasma was centrifuged at 4 °C for 5 minutes at 12 000 × g. The supernatant was collected and transferred to a new tube, 5 µl was immediately analysed using an ISCUSS Analyser (CMA Microdialysis). The detection limit was 1.0 to 150 µmol/L.

### Enzyme-linked immunosorbent assay

SP quantitation was performed by using an enzyme-linked immunosorbent assay kit from Enzo Life Sciences (ADI-900–018, Farmingdale, NY). Saliva and plasma samples were prepared and analysed according to the manufacturer’s recommendations.

### Capillary Isoelectric Focusing (CIEF) immunoassay

Saliva samples were centrifuged to remove debris and the supernatants were extracted to a new tube. Thereafter the samples were diluted/buffer-exchanged with Bicince/Chaps, concentrated and desalted using Amicon® Ultra centrifugal filters (Merck Millipore, Billericia, MA, USA). Total protein of saliva was measured with 2D-Quant kit according to user manual (GE Healthcare, Little Chalfont, UK.) Plasma samples were subjected to albumin and IgG removal kit (GE Healthcare) and then concentrated and desalted as described above. The samples were analysed using a charge-based assay (saliva) and size-based assay (plasma) using capillary IEF with Peggy system (ProteinSimple, Santa Clara, Ca, USA) according to the user manual. Briefly, samples were analysed with fluorescent *pI* standard 3–10 or fluorescent molecular weight markers 12–230 kDa. A protein concentration of 1 mg/ml and 0.5 mg/ml was used for analysis of BDNF and NGF/CGRP respectively. The proteins were detected using primary mouse BDNF (Abcam, ab10505, Cambridge, UK, dilution 1:50), rabbit NGF (Abcam, ab6199, Cambrige, UK, dilution 1:25) and rabbit CGRP (Abcam, ab189786, Cambridge, UK, dilution 1:25). Proteins were separated based on charge or size in the capillary by applying currents. Signal was detected with Luminol and Peroxide and scanned with a charge-coupled device camera. Data generated was analysed in compass software version 2.7.1 (ProteinSimple, Santa Clara, Ca, USA).

### Statistics

Differences between males and females in the study were tested with Mann-Whitney U-test since most variables did not show normal distribution. Repeated measurement analysis of variance (ANOVA) was used to analyse differences in salivary flow between collection methods and Bonferroni was used as post hoc test when the ANOVA indicated significant differences. To analyse the concentrations of neuropeptides in different saliva samples repeated measures using Friedman’s test and additional post-hoc analysis with Wilcoxon matched pair-test and Bonferroni adjustment was used.

Correlations between variables were tested for statistical significance with Spearman correlation test, adjusted for multiple comparisons according to Bonferroni. Intra-class coefficients between biomarker expression in different types of saliva and plasma were calculated using Excel Analysis tool pack. Descriptive data are presented as mean ± SD or median and interquartile range (IQR). For all analyses, the significance level was set at P < 0.05.

The statistical analyses were performed using Statistica version 13 (StatSoft, Oklahoma, USA).

## Results

### Data overview

Descriptive data of participants in the study are presented in Table 1. Participants included in the study showed no signs of psychological distress. The women in the cohort however, reported slightly higher values in the total number of teeth, somatic and anxiety symptoms (P < 0.01).

**Table 1:** Overview of the participants in the study. Questionnaire scores are described as means ± standard deviation or as median (interquartile range). Statistical parameters are only reported when the distributions in the two groups differed significantly, P < 0.05 (Mann–Whitney U-test).

Variable	Males (n = 10)	Females (n = 10)	Statistics
Age (Years)	24.7 ± 3.1	24.9 ± 3.3	
Body Mass Index (kg/m^2^)	23.4 ± 3.0	22.5 ± 3.0	
Number of teeth	29 (2)	32 (2)	*U* = 18.5; *P* < 0.01
**Salivary Flow (ml/min)**			
Unstimulated whole saliva	0.196 ± 0.082	0.185 ± 0.065	
Unstimulated sublingual saliva	0.104 ± 0.060	0.317 ± 0.640	
Stimulated parotid saliva	0.215 ± 0.155	0.20 ± 0.290	
Stimulated whole saliva	1.889 ± 0.473	2.121 ± 1.181	
PHQ-9 Score	0 (1)	1 (3)	
PHQ-15 Score	0 (0)	1.5 (2)	*U* = 5.0; P < 0.001
GAD-7 Score	0 (0)	1 (1)	*U* = 13.5; P < 0.01
PSS-10 Score	5.5 (4)	8 (4)	
JFLS Score	0 (0)	0 (0)	

n = number of subjects in each group; PHQ = The Patient Health Questionnaire; GAD = Generalized Anxiety Disorder; PSS = perceived stress scale; JFLS = Jaw Functional Limitation Scale.

Salivary flow differed significantly between collection methods but was not affected by sex (P > 0.05). Stimulated whole saliva showed higher flow rate (2.0 ± 0.9 ml/min) compared to other collection methods (P < 0.001).

### Nerve growth factor

**Figure 2:**
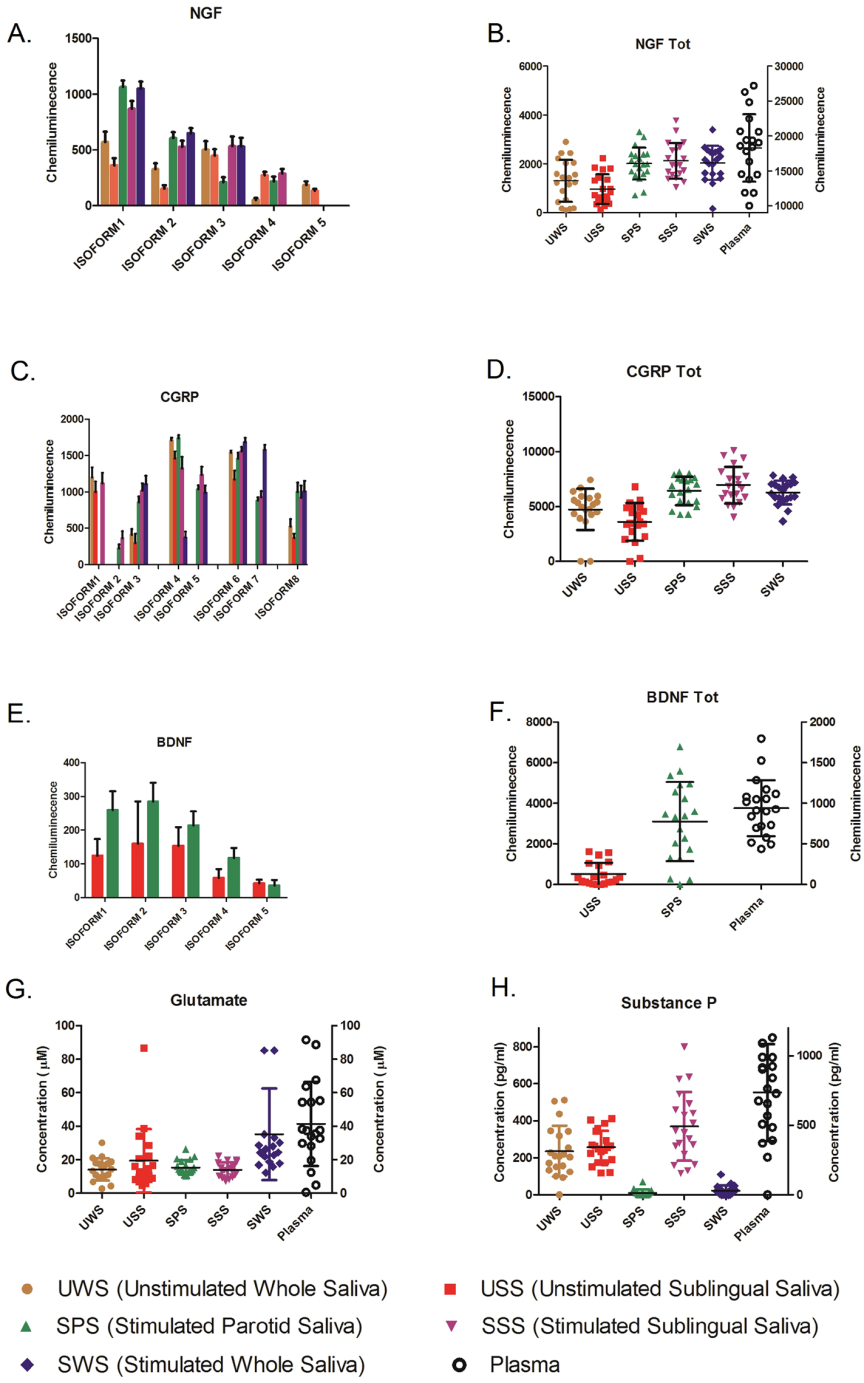
Salivary and plasma NGF, CGRP, BDNF, glutamate and substance P concentration in 20 healthy individuals matched for age and gender (Fig. 2A–H). Large variations were observed between different collection methods. Several isoforms was detected for NGF, CGRP and BDNF. The isoform pattern showed significant variation in both expression and chemiluminescence levels between different collection methods (Friedman; P < 0.05). All stimulated saliva samples, whether chemically or mechanically stimulated, showed significant higher expression of total-NGF and total-CGRP compared to unstimulated saliva samples and plasma. Higher concentration of glutamate was found in stimulated whole saliva comparing to other salivary collection methods. However, the plasma levels of glutamate and substance P were significantly higher in comparison with the levels detected in saliva.

Five different isoforms, in the *pI* range of 5.6 to 7.0, were detected for NGF in saliva (Fig. 2A). There were significant differences between saliva types in the chemiluminescence of NGF-1 (*pI* 5.6) (X^2^ = 25.1; P < 0.001) and NGF-2 (*pI* 6.0) levels (X^2^ = 15.5; P < 0.01). Both isoforms showed significantly higher expression in stimulated parotid and whole saliva in comparision to unstimulated sublingual and whole saliva (P < 0.05). Stimulated sublingual saliva did not differ regading NGF-1 and NGF-2 levels compared to other collection methods (Fig. 2A).

NGF-3 (*pI* 6.2) showed no differences in chemiluminescence between sampling methods (X^2^ = 7.5; P > 0.05). NGF-4 (*pI* 6.5) could not be detected in stimulated whole saliva but was frequent occuring in the four other saliva types (X^2^ = 27.9; P < 0.001). The expression of NGF-4 was decreased in unstimulated whole saliva (53.8 ± 77.2) compared to the other collection methods (265 ± 174 P < 0.05). NGF-5 (*pI* 7.0) was only detected in the two unstimulated saliva samples, however, no significant differences in expression level was found (X^2^ = 0.8; P > 0.05)

Total NGF level in unstimulated whole saliva (1,313 ± 860) and sublingual saliva (966 ± 609) showed lower expression compared to the other collection methods (Fig. 2B). All stimulated saliva samples, whether chemically or mechanically stimulated, showed significant higher expression of total NGF compared to unstimulated saliva samples (X^2 = ^35.2; P < 0.001). Plasma levels (17,376 ± 6,242) showed the highest expression of total NGF in comparison to all saliva-sampling methods (Z = 3.9; P < 0.001).

Levels of total NGF in stimulated sublingual saliva and stimulated whole saliva correlated (r_s_ = 0.70, P < 0.001), no other correlations were found. The ICC between levels in saliva and plasma was 0.003.

### Calcitonin gene-related peptide

CGRP was detected in eight different isoforms in the *pI* range of 4.6 to 6.9 (Fig. 2C). The different isoforms showed great variation in the expression and occurrence between different collection methods. CGRP-3 (*pI* 5.8), CGRP-6 (*pI* 6.4) and CGRP-8 (*pI* 6.9) could be detected in all saliva types, while the other isoforms (CGRP-1; *pI* 4.6, CGRP-2; *pI* 5.6, CGRP-4; *pI* 6.1, CGRP-5; *pI* 6.2, CGRP-7; *pI* 6.5) only could be detected in some saliva types, especially in stimulated sublingual saliva. These former CGRP isoforms showed a tendency towards higher expression in stimulated saliva samples compared to unstimulated saliva samples. CGRP-3, for example, was significantly more expressed in stimulated sublingual (1,232 ± 421) and parotid saliva (1,139 ± 176) compared to unstimulated whole saliva (605 ± 374; Z = 3.6, P < 0.001). Similar expression tendency was also observed for CGRP-8 (X^2^ = 28.0; P < 0 0.001).

Stimulated saliva expressed higher total CGRP compared to unstimulated saliva (X^2^ = 4.6; P < 0.001), but the difference was only significant for stimulated sublingual saliva (6,944 ± 1,669; Z = 3.7, P < 0.001). There were no significant differences in total CGRP expression between unstimulated whole (4,729 ± 1,872) and sublingual (3,596 ± 1,737; Z = 2.3, P < 0.05) saliva.

In plasma, CGRP was not possible to detect correctly with the developed protocol.

Total CGRP levels in stimulated sublingual saliva correlated with total CGRP levels in stimulated whole saliva (r_s_ = 0.78, P < 0.001). The ICC between levels in saliva and plasma was 0.150.

### Brain-derived neurotrophic factor

BDNF was found in saliva in five different isoforms, in the *pI* range of 4.6 to 4.7 (Fig. 2E), but detected in only unstimulated sublingual saliva (513 ± 565) and stimulated parotid saliva (3,097 ± 1,944) with significant higher expression in latter (Z = 3.7; P < 0.01).

In unstimulated sublingual saliva no significant differences was observed between the isoforms. However, in stimulated parotid saliva BDNF-1 was most frequenly expressed (X^2^ = 14.2; P < 0.01). Stimulated parotid saliva also showed significantly higher expression of total BDNF compared to plasma (942 ± 345) (Z = 3.4; P < 0.001).

There were no significant correlations between total BDNF levels in saliva or plasma. The ICC between levels in saliva and plasma was 0.001.

### Glutamate

The glutamate level (Fig. 2G) showed large variations between the five different saliva collection methods (X^2^ = 30.3; P < 0.001). Post-hoc analysis showed significantly higher levels of glutamate in stimulated whole saliva (34.2 ± 26.1 µg/l) comparing to other salivary collection methods (Z = 3.8; P < 0.001).

The plasma level of glutamate was higher (39.4 ± 26.1 µg/l) compared to saliva (X^2 = ^37.1; P < 0.001). However, significant differences in the glutamate levels between plasma and saliva samples were only detected for unstimulated whole and sublingual saliva (Z = 2.9; P < 0.01) after Bonferroni adjustment for multiple comparisons. The glutamate levels in stimulated whole saliva were similar to the plasma concentration (Z = 1.6; P > 0.05).

Total glutamate levels in stimulated sublingual saliva correlated to total glutamate levels in resting whole (r_s_ = 0.64, P = 0.003) and resting sublingual saliva (r_s_ = 0.63, P = 0.003). There was also moderate, but insignificant correlations between total glutamate in stimulated whole saliva and resting sublingual saliva (r_s_ = 0.56, P = 0.010) as well as between the levels in stimulated whole saliva and plasma (r_s_ = 0.56, P = 0.011) after Bonferroni adjustment for multiple comparisons. The ICC between levels in saliva and plasma was 0.165.

### Substance P

The SP level also showed large variations between the five different saliva collection methods (X^2^ = 54.6; P < 0.001) (Fig. 2H). Post-hoc analysis revealed significantly higher levels of SP in saliva derived mainly from the sublingual and submandibular glands e.g. whole unstimulated saliva (235 ± 137 pg/ml), unstimulated (257 ± 89 pg/ml) and stimulated sublingual saliva (370 ± 185 pg/ml) when comparing to saliva high in parotid content e.g. whole stimulated saliva (23 ± 27 pg/ml) and parotid saliva (11 ± 17 pg/ml).

Further, significant variations were also detected between the salivary and plasma levels of SP (X^2 = ^69.0; P < 0.001) with significantly higher levels of SP in plasma (737 ± 349 pg/ml) compared to all saliva collection methods (P < 0.01).

There were no significant correlations between total SP levels in saliva or plasma. The ICC was 0.003.

### Correlations between biomarker levels

There were significant positive correlations between levels of total CGRP and NGF in resting (r_s_ = 0.67, P < 0.002) and stimulated whole (r_s_ = 0.59, P < 0.006) as well as in stimulated sublingual saliva (r_s_ = 0.57, P < 0.008).

## Discussion

In the current study, five biomarkers related to pain were investigated in different types of saliva and plasma. With new sensitive technology based on western blot we are first to find several isoforms of NGF, CGRP and BDNF in saliva. The expression showed great variations between different saliva collection methods, as shown by the general lack of significant correlations and low ICCs in levels between the different methods. The expression of NGF and BDNF showed association with salivary flow while SP for example was associated with the glandular origin of the saliva. Glutamate was mostly expressed in whole stimulated saliva and in contrast to the other biomarkers, the concentration was moderately correlated between saliva types as well as in plasma. However, the ICC was still very low. Taken together this means that the methods are not interchangeable, and comparisons across studies can only be made if the same collection method is used.

We are first to show that the glutamate concentration in saliva is associated with the method used for sample collection with the highest levels found in stimulated whole saliva. The concentration in stimulated whole saliva was also moderately correlated to the plasma levels, which consequently could imply a less invasive alternative to blood collection. Evidence from the last decade indicates that glutamate has an important role in nociceptive processes. In the periphery, glutamate receptors have been identified on nociceptive nerve terminals in the skin and muscles^20,21^, which makes it likely that glutamate would interact with these receptors. The concentration of glutamate has also been shown to be elevated in different pain conditions^22,2,4,19^. These findings indicate that glutamate may be an essential pain mediator in peripheral tissue and may therefore act as a potential pain biomarker among others.

BDNF is another factor that has been recognized as a modulator of the nociceptive pathways. In our study BDNF was found at *pI* 4.66 to 4.71 as five different bands, which indicates expression of different isoforms. These results show similarity to an earlier study were pro and mature BDNF was identified in whole saliva using western-blot analysis^11^. However, in contrast to that study, in our study expression of BNDF could only be detected in unstimulated sublingual saliva and stimulated parotid saliva. The reason for the differences across studies could be due to different methods for analysis, the strictly controlled patient sample in our study, and perhaps also the relatively small sample size in both studies. The levels found in saliva did not reflect systemic BDNF levels and are therefore BDNF is unlikely to be useful as a diagnostic tool, as has been described earlier^23^.

Salivary SP was first identified by Parris *et al*.,^24^. The authors showed that SP was decreased in whole saliva among patients with chronic back pain compared to controls, and higher concentration was observed in whole saliva compared to plasma using radioimmunoassay^24^. This is contradictory to our results were ELISA was used and the opposite relation was shown with significant higher levels of SP in plasma compared to all saliva collection methods. Nevertheless, the plasma SP showed large individual variations and the salivary SP showed large variations between different saliva collection approaches. It seems to be significantly more concentrated in saliva mainly derived from the glands situated sublingually when comparing to saliva high in parotid content. The submandibular glands are by far the most active in the unstimulated resting state, and they are estimated to produce about 65% of the total resting volume. However, when salivary glands are stimulated the parotid gland can account for more than 50% of the whole saliva volume in the mouth^25,26^. Several animal studies have shown that the submandibular gland have SP containing sensory fibres which may explain the higher concentration of SP in saliva derived from these glands. It has also been shown that a small part of the SP-like immunoreactivity found in the submandibular gland is of sensory origin, whereas the majority appears to be intrinsic^27^.

A limited number of studies have been performed on NGF in saliva. Nam and co-authors for example measured NGF in three sources of saliva and found similar levels to our study^5^. They found also an association between the NGF concentration and age in stimulated submandibular saliva, and further between gender and NGF concentrations in unstimulated whole and stimulated parotid saliva. Whereas a recent study has suggested marginal differences in several inflammatory cytokines between young and elderly participants. In our study however, age could not further be examined because of the limited age-interval^28^. Nevertheless, we could demonstrate a significant association between stimulation of salivary flow and NGF expression. This since all stimulated saliva samples, whether chemically or mechanically stimulated, showed significant higher expression of total NGF compared to unstimulated saliva samples and plasma. This is in agreement with an earlier study were NGF concentration also was shown to be significantly increased with salivary flow^29^. In our study this tendency was also observed for salivary CGRP, but the data was only significant for stimulated sublingual saliva.

When analysing NGF, CGRP and also BDNF automatized Western-blot was used. This approach has the advantage to detect and quantify proteins/peptides based on charge. NGF was detected in five different isoforms with this new sensitive method. Olausson *et al*.^30^ have reported three isoforms of NGF using two dimensional gel electrophoresis. The *pI*s for the isoforms that we have identified in this study are in accordance with the *pI* range reported by them^30^. CGRP was found in eight different isoforms in saliva. The expression of each of these isoforms was highly affected by the method for collection. Sample methodology is thus an important factor to consider when studying CGRP and NGF in saliva. We were able to identify different isoforms that could be due to post translational modifications such as phosphorylation. According to the protein database Uniprot (expasy.org) there are potential sites of phosphorylation in the amino acid sequences of these proteins that leads to the different *pI* of the proteins. Further studies for protein characterization of these isoforms are needed, to be able to conclude the distribution and importance of the isoforms in the different saliva glands.

Salivary flow showed alternations in accordance with previous reported levels^31–33^. As presented in Table 1 data showed that the method of collection directly affects salivary flow. Lowest salivary flow rate was not surprisingly observed for glandular saliva and the highest flow rate was observed for stimulated whole saliva.

When searching for the optimal collection method to study salivary pain biomarkers, there are advantages and limitations with all the different methods that should be considered. Glandular saliva is specific and pure but requires specific devices and blockage of saliva from the unrequired glands during collection. This may result in longer collection sessions that may be affected by internal and external factors. Regarding sublingual saliva, it is well-known that the submandibular and sublingual glands are anatomically closely located (Fig. 1), and can sometimes share the same ducts which is the reason why we in the current study choose to sample saliva from both glands simultaneously^34^.

Whole saliva is however easy to control and accesses, and showed high levels of all analytes, except SP and BDNF. In earlier studies by our group it has also been shown that this method expressed the least variability (CV 35%) regarding protein expression^13^. Thus, based on our results, we suggest the use of stimulated whole saliva for analyses of salivary biomarkers. This is based on the simplicity of the method, low variability and significantly higher expression of several markers. The consistency when collecting saliva is however of greater importance than the method itself, as is shown in Fig. 2. Further research is needed to evaluate these markers and specifically to studying these markers in different pain processes. This study should be regarded as one brick to further research evaluating saliva as a medium to study different biomarkers related to pain.

In most previous studies regarding pain biomarkers plasma has been the analytical medium and only a handful previous studies have assessed the saliva concentration^7–9,11,23^. As described earlier there are several advantages using saliva over blood. However, a common denominator in studies on saliva is the lack of standardized techniques for saliva sample collection and proper characterization of the patients. Since saliva is not a single fluid, these aspects make it nearly impossible to compare studies because of the great variability. Hence, to our knowledge this is the first study that structurally have compared the expression of pain related mediators in a strictly controlled healthy cohort.

The samples were structurally collected from twenty individuals during the same time and conditions. In order to reduce the influence of external factors on salivary flow and secretions the inclusions criteria were very rigid. It was ensured via the patient history and the careful oral and dental examination that the participants were healthy. From all participants, both glandular and whole saliva was collected. These were collected during rest and chemical as well as mechanical stimulation.

Some considerations, however, need to be addressed. It was ensured via the anamnesis that the female subjects were not pregnant, lactating or using any contraceptives to reduce the influence of hormonal variations. Since it is not clear whether the menstrual cycle affects the salivary response and salivary flow we did not control for menstrual cycle variations. Further, according to some findings age may affects protein expression^35^ but a recent study have shown negligible differences in inflammatory markers in young and elderly individuals^28^. A limited age interval was however used in the current study design to minimise a possible confounder. This may limits its external validity.

In conclusion, this is the first study to (1) detect NGF, CGRP, BDNF, glutamate and SP in five different salivary types (2) develop a new protocol/method for analysis of different isoforms of NGF, CGRP and BDNF (3) show measurable levels of several isoforms of NGF, CGRP and BDNF in human saliva, and finally 4) demonstrate a correlation between the glutamate level in stimulated whole saliva and plasma.
